# Mandible-Specific Noise Reduction in Pediatric Dental CBCT with Simulated Noise: Search Window Selection for the Fast Non-Local Means Algorithm

**DOI:** 10.3390/life16101645

**Published:** 2026-09-30

**Authors:** Hee-Seo Kim, Hajin Kim, Young-Eun Kwon, Ji-Youn Kim

**Affiliations:** 1 Department of Dental Hygiene, Gachon University, 191, Hambangmoe-ro, Yeonsu-gu, Incheon 21936, Republic of Korea; gmltj127@gachon.ac.kr; 2 Department of Health Science, General Graduate School of Gachon University, 191, Hambangmoe-ro, Yeonsu-gu, Incheon 21936, Republic of Korea; happida3@gachon.ac.kr; 3 Department of Oral and Maxillofacial Radiology, School of Dentistry, IHBR, Kyungpook National University, 2175, Dalgubeol-daero, Jung-gu, Daegu 41940, Republic of Korea

**Keywords:** cone-beam computed tomography, fast non-local means algorithm, search window, mandible, noise reduction, pediatric patients

## Abstract

Accurate assessment of the pediatric mandible using dental cone-beam computed tomography (CBCT) is crucial despite radiation exposure concerns. The fast non-local means (FNLM) algorithm reduces noise in CBCT images, but its performance depends on parameter selection. This study compared FNLM search window sizes for pediatric mandibular CBCT images under simulated noise degradation. Mandibular CBCT images were acquired from 17 pediatric datasets in the MMDental database. Noise degradation was simulated by adding Poisson and Gaussian noise (σ = 0.05). The FNLM algorithm was applied using search windows from 3 × 3 to 21 × 21 pixels and image quality was assessed using noise, similarity, and no-reference metrics and visual evaluation. Most metrics changed progressively with increasing search window size, with smaller changes beyond 7 × 7, and BRISQUE reached its lowest value at 7 × 7. Visual assessment showed noise reduction but decreased sharpness of anatomical boundaries with larger search windows. Among the evaluated conditions, the 7 × 7 search window provided the best compromise between noise reduction and structural preservation. These findings indicate that appropriate search window selection is important for FNLM-based noise reduction in pediatric mandibular CBCT images and should be interpreted within the simulated conditions of this study.

## 1. Introduction

The cranial-to-facial proportion changes significantly during development, decreasing from approximately 8:1 at birth to 2.5:1 in adulthood [1,2,3]. As the facial skeleton grows in an anteroinferior direction, facial structures become increasingly prominent and less protected by the cranial base, thereby increasing susceptibility to trauma. Consequently, children and adolescents experience a high incidence of maxillofacial injuries, with the mandible being the most common site of fractures [1,2,3,4,5,6,7,8]. Depending on the site of impact, pediatric mandibular fractures are frequently accompanied by fractures of the condyle or mandibular angle. Furthermore, the wide marrow space, thin cortical bone, and developing permanent tooth buds characteristic of pediatric mandible complicate radiographic assessment. Although adequate visualization of mandibular structures is important given the ongoing growth of the pediatric craniofacial skeleton, developing dentition and immature facial structures can obscure fine anatomical details and reduce image interpretability. Consequently, three-dimensional (3D) imaging modalities, such as dental cone-beam computed tomography (CBCT), are widely used to improve diagnostic accuracy in pediatric maxillofacial trauma.

Although CBCT provides 3D volumetric information that overcomes the superimposition and distortion inherent in conventional two-dimensional imaging, this diagnostic advantage generally comes at the cost of higher radiation exposure compared to conventional radiographic techniques [9,10,11,12]. Consequently, various low-dose imaging protocols have been proposed to minimize radiation exposure in pediatric patients, a population that is particularly susceptible to radiation owing to rapid tissue growth and active cellular proliferation. However, reducing the radiation dose often degrades image quality, which can compromise the diagnosis and treatment planning of pediatric maxillofacial trauma. Recently, the paradigm of radiation protection has shifted from maintaining radiation exposure as low as reasonably achievable to maintaining radiation exposure as low as diagnostically acceptable through an indication-oriented and patient-specific approach [9,13]. Under this paradigm, ensuring image quality sufficient for diagnosis has become an important consideration, particularly in pediatric maxillofacial trauma, where accurate evaluation of fine anatomical structures is required. As a result, relatively high exposure settings are still employed in pediatric maxillofacial trauma imaging. Therefore, instead of increasing radiation dose to improve diagnostic performance, alternative strategies are needed to minimize radiation-related risk while maintaining diagnostic accuracy under low-dose conditions [14].

Various image enhancement techniques have been proposed to compensate for image quality degradation under reduced-dose CBCT conditions. Among them, the non-local means (NLM) algorithm has attracted considerable attention, as it can be directly applied without prior training and effectively preserves anatomical structures through patch-based similarity estimation. The NLM algorithm assigns weights based on similarity rather than physical distance, enabling effective noise reduction while preserving anatomical boundaries and fine structural details [15,16,17,18]. These characteristics are particularly advantageous for dental images, which contain substantial anatomical self-similarity arising from the repeated representation of teeth and surrounding osseous structures. However, the conventional NLM algorithm requires repetitive similarity calculations for many image patches, resulting in substantial computational cost and limited processing efficiency. To address this limitation, the fast NLM (FNLM) algorithm was developed using acceleration strategies such as discrete convolution and summed squared intensity maps, substantially reducing computational complexity while maintaining noise reduction performance. Despite these advantages, the performance of the FNLM algorithm is highly dependent on the selection of appropriate parameters [19,20].

The search window size is a critical parameter that determines the trade-off between noise reduction and structural preservation. A small search window limits the ability to identify structurally similar patches, thereby reducing the exploitation of non-local information and ultimately degrading noise reduction performance. In contrast, an excessively large search window improves noise reduction through weighted averaging; however, it also increases the likelihood of averaging anatomically distinct structures with similar intensity patterns, thereby reducing fine structural details and boundary sharpness. Appropriate selection of the search window size is therefore essential for achieving effective noise reduction while preserving diagnostically important anatomical features. These considerations suggest that FNLM represents a promising approach for enhancing image quality while maintaining anatomical fidelity in noise-degraded pediatric CBCT images. Nevertheless, studies investigating the selection of FNLM search window size for improving image quality in noise-degraded CBCT images, particularly in the mandibular region of pediatric patients, remain limited. Because the mandible is a frequent site of maxillofacial fractures in pediatric trauma, it was selected as the anatomical region of interest in the present study. As a preliminary step toward the application of FNLM to pediatric mandibular fracture cases, this study first evaluated the image quality improvement performance of FNLM in the mandibular region under simulated noise degradation. This approach may provide a basis for further application of FNLM in dental imaging by evaluating its ability to effectively reduce noise while preserving fine anatomical structures. Therefore, this study applied the FNLM algorithm to pediatric CBCT images degraded by simulated noise and compared search window sizes to identify the condition that provided the best overall compromise between noise reduction and preservation of fine anatomical structures in the mandibular region.

## 2. Materials and Methods

### 2.1. Acquisition of CBCT Data

Dental CBCT data were obtained from the MMDental dataset, which contains records from 660 patients aged 5–86 years [21]. All patients had medical records, and CBCT data were also available for 403 of them. Within the MMDental dataset, CBCT images were acquired using either the HiRes 3D-Plus system (Beijing LargeV Instrument Co., Ltd., Beijing, China) or oral and maxillofacial CBCT equipment (Changzhou Boen Zhongding Medical Technology Co., Ltd., Changzhou, China). Both systems provided volumetric images with dimensions of 640 × 640 × 400 voxels, and a slice thickness of 0.25 mm. For pediatric patients, reduced radiation dose protocols were applied relative to those used for adult patients, with scanning parameters of 100 kVp/3 mA for the HiRes 3D-Plus system and 90 kVp/6 mA for the oral and maxillofacial CBCT equipment.

A total of 17 pediatric patients aged 18 years or younger were selected from the 403 patients with available CBCT data. The selected patients comprised 6 males and 11 females, with a mean age, 14.6 ± 4.7 years (range, 5–18 years). To select data suitable for evaluating the entire dental arch, one mandibular axial slice per patient was manually selected by a single researcher using MATLAB (ver. R2025b; MathWorks, Natick, MA, USA). Based on basic knowledge of dental and mandibular anatomy acquired through dental hygiene education, the researcher selected a slice containing a sufficient mandibular area for placement of the minimum regions of interest (ROIs) required for image quality assessment while including the tooth region.

### 2.2. Simulation of Noise Degradation

To simulate noise degradation, Poisson and Gaussian noise were added to the selected slices. The original CBCT images may contain inherent noise and artifacts and were used as reference images rather than noise-free ground truth. The acquisition parameters of the publicly available CBCT dataset differed from those commonly used in adults primarily by a reduction of approximately 2 mA in tube current, while similar tube voltage settings were maintained. Because this reduction was considered insufficient to produce a clearly degraded imaging condition, additional noise was added to the images to evaluate the noise reduction performance of the FNLM algorithm.

**Figure 1:**
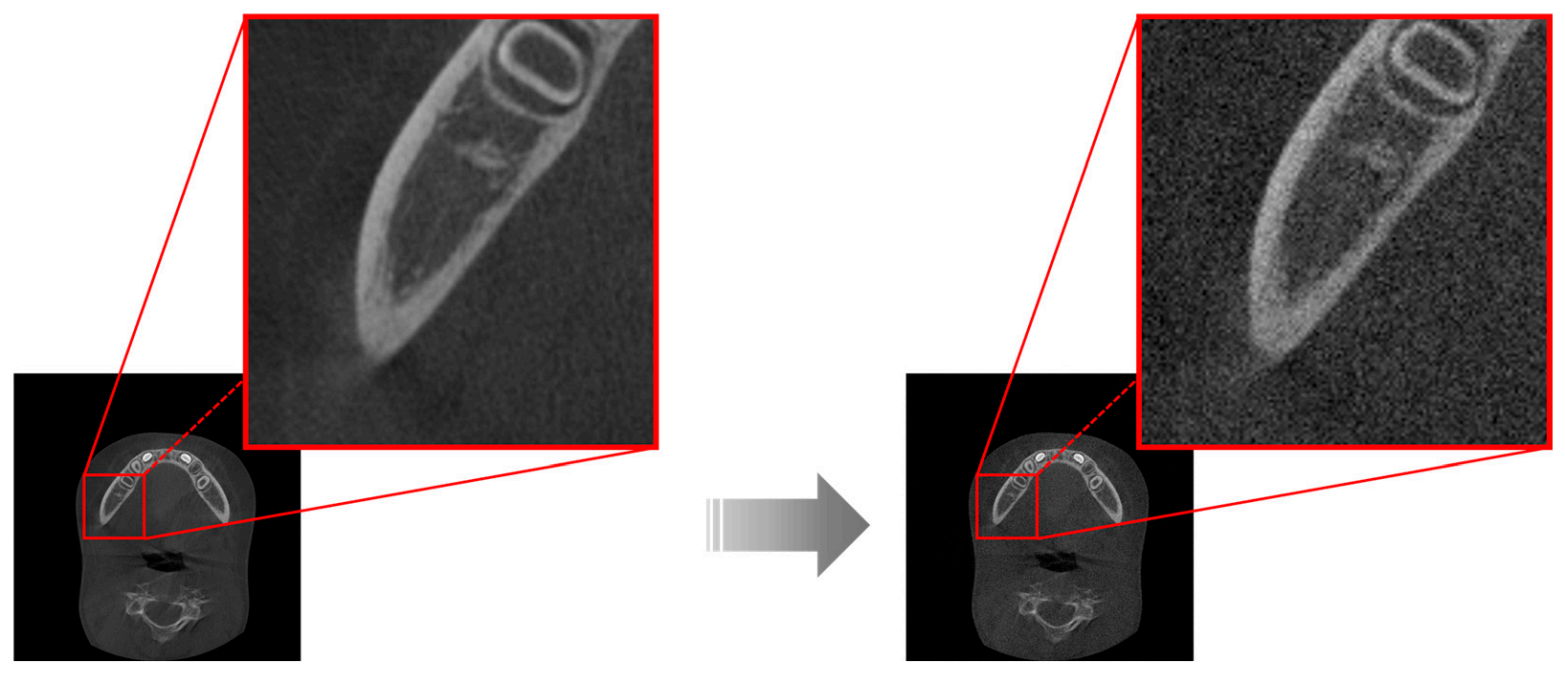
Simulated noise degradation of dental cone-beam computed tomography (CBCT) images through the addition of Poisson and Gaussian noise. The red box indicates the mandibular region shown at higher magnification to facilitate visual comparison.

In CBCT, image formation involves the generation of X-ray photons, their transmission through the subject, and subsequent detection by the detector system. During this process, quantum noise arises from stochastic fluctuations in the detection of X-ray photons, whereas electronic noise originates from the detector and data acquisition system [22,23]. Accordingly, Poisson noise was intended to model signal-dependent quantum noise, and Gaussian noise was added to represent additive electronic noise that can be modeled independently of the signal. Specifically, each selected slice was extracted from the NIfTI volume and converted to an 8-bit grayscale image (intensity range, 0–255) by min–max normalization of the slice, and was then converted to double precision and normalized to the range [0, 1] by dividing by 255 prior to noise addition. Poisson noise was first applied to the normalized double-precision image using the imnoise function of the MATLAB Image Processing Toolbox (‘poisson’ option), and zero-mean Gaussian noise with an empirically selected standard deviation of *σ* = 0.05 on the normalized [0, 1] scale was subsequently added using the randn function, after which the image was clipped to [0, 1] and converted back to an 8-bit image (Figure 1). Because the imnoise function scales double-precision input by 10^12^ when generating Poisson-distributed values, the Poisson component generated in this implementation was negligible (on the order of 10^−7^ on the [0, 1] scale), and the resulting degradation was predominantly Gaussian. A single noise realization was generated for each image without a fixed random seed and used for all search window conditions.

The Gaussian noise level was not intended to directly represent the actual noise magnitude associated with a specific clinical low-dose CBCT protocol. Rather, *σ* = 0.05 was selected as a simulation parameter to provide a sufficiently degraded imaging condition for evaluating the noise reduction capability and structural preservation performance of the FNLM algorithm. In particular, this level was selected to produce clearly observable noise-induced image degradation in the mandibular region while maintaining recognizable structural information in both cortical and trabecular bone.

### 2.3. Modeling of the FNLM Algorithm

To address the high computational cost of the conventional NLM algorithm, FNLM incorporates acceleration strategies based on discrete convolution and summed squared intensity maps. Discrete convolution enables rapid estimation of patch cross-correlation through matrix-based computation, while the summed squared intensity map minimizes redundant calculations by utilizing precomputed patch intensity values. Such acceleration strategies substantially reduce computational complexity and improve computational efficiency when processing large CBCT datasets. The mathematical formulation of the FNLM algorithm follows the conventional NLM weighting framework, with computational acceleration of similarity calculation implemented as follows [24]:

(1) $$\mathrm{NL}\left[I\right]\left(p\right)=\;\sum_{q\in I}\omega \left(p,q\right)I(q)$$

where *p* and *q* denote the target pixel and a candidate pixel within the search window, respectively, and *ω*(*p*,*q*) represents the similarity weight:

(2) $$\omega \left(p,q\right)=\;\frac{1}{Z(p)}\exp\left(-\frac{\sum_{\delta }G_{\sigma_{k}}(\delta ){\left\|I\left(p+\delta \right)-I(q+\delta )\right\|}_{2}^{2}}{d^{2}}\right)$$

where *δ* represents the two-dimensional (2D) spatial offset within the local patches centered at pixels *p* and *q*, respectively, and $G_{\sigma_{k}}\left(\delta \right)$ denotes the Gaussian kernel with standard deviation *σ_k_* applied to the corresponding patch offset. The term ${\left\|I\left(p+\delta \right)-I(q+\delta )\right\|}_{2}^{2}$ indicates the squared intensity difference between corresponding locations within the two patches, and *Z*(*p*) is a normalization constant.

(3) $$Z\left(p\right)=\sum_{q}\exp\left(-\frac{\sum_{\delta }G_{\sigma_{k}}(\delta ){\left\|I\left(p+\delta \right)-I(q+\delta )\right\|}_{2}^{2}}{d^{2}}\right)$$

The FNLM algorithm reduces computational complexity by vectorizing the 2D image into a one-dimensional (1D) sequence. In the vectorized representation, the relative displacement between the target pixel *p* and candidate pixel *q* is defined as *τ* = *q* − *p*. Thus, corresponding locations in the target and candidate patches can be represented by *k* and *k* + *τ*, respectively, where *k* = *p* + *δ* denotes the 1D index within the vectorized image and *δ* ∈ [−*P*, *P*] is the 1D counterpart of the patch offset in Equations (2) and (3). In the 1D formulation, the Gaussian weighting $G_{\sigma_{k}}(\delta )$ is replaced by uniform weighting over the patch ($G_{\sigma_{k}}\left(\delta \right)=1$), so that the patch distance in Equation (2) reduces to a plain sum of squared intensity differences over a 1D window of length 2*P* + 1. This transformation allows the squared intensity differences between corresponding patch locations to be accumulated along the 1D sequence.

(4) $$H_{\tau }\left(s\right)=\sum_{k=0}^{s}{\left\|I\left(k\right)-I(k+\tau )\right\|}_{2}^{2}$$

Accordingly, the patch distance over the window [*p* − *P*, *p* + *P*] is obtained as the difference of two cumulative sums, and the patch similarity term can be expressed as

(5) $$\omega \left(p,q\right)=\frac{1}{Z(p)}\exp\left(-\frac{H_{\tau }\left(p+P\right)-H_{\tau }(p-P-1)}{d^{2}}\right)$$

where *s* denotes the position within the vectorized 1D representation, *H_τ_*(*s*) denotes the cumulative sum of squared intensity differences for the displacement *τ*, and *P* represents the half-width of the corresponding 1D patch, and $Z\left(p\right)=\sum_{\tau }\exp\left(-\frac{H_{\tau }\left(p+P\right)-H_{\tau }(p-P-1)}{d^{2}}\right)$ is the normalization constant.

Compared with the conventional NLM, FNLM improves computational efficiency through accelerated similarity calculations, while retaining the fundamental non-local weighting framework. However, despite this substantial reduction in computational complexity, FNLM noise reduction performance remains highly dependent on the selection of appropriate parameters. In particular, the search window size is a key parameter affecting the balance between noise reduction and preservation of anatomical structures.

In light of the above, this study investigated the effect of search window size on both noise reduction performance and preservation of mandibular anatomical structures to identify the search window size providing the best overall compromise between these competing factors. Search window sizes ranging from 3 × 3 to 21 × 21 pixels were adjusted at intervals of 2 × 2 pixels, resulting in a total of 10 conditions. To isolate the effect of search window size, the patch size and smoothing factor were fixed at 7 × 7 and 0.05, respectively, based on empirical selection.

The noise simulation, FNLM processing, and processing-time measurements were all performed in MATLAB (ver. R2025b) on a workstation running Windows 11 Education (64-bit) with an Intel Core Ultra 7 265K processor (3.90 GHz) and 32 GB of RAM. The FNLM algorithm was implemented as a custom MATLAB function and executed on the CPU; although the workstation was equipped with an NVIDIA GeForce RTX 5080 GPU (16 GB), no GPU acceleration was used.

### 2.4. Quantitative Image Quality Evaluation

To evaluate the noise reduction performance of each search window condition, quantitative image quality assessment was performed using three categories of metrics: noise level evaluation metrics, similarity evaluation metrics, and no-reference image quality assessment metrics [24,25,26,27].

#### 2.4.1. Noise Level Evaluation Metrics

**Figure 2:**
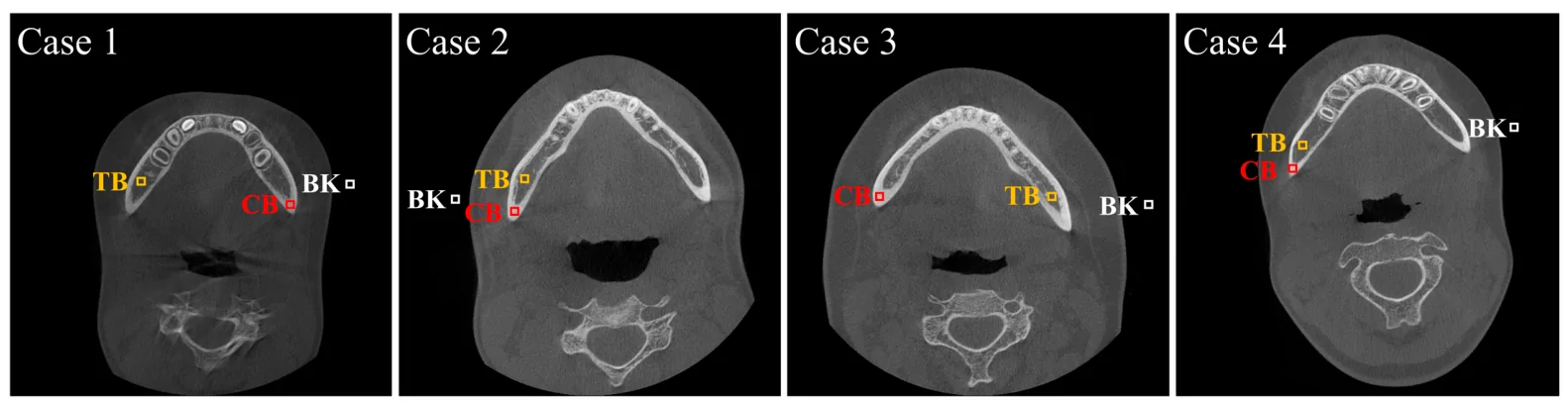
Representative axial CBCT images showing the placement of cortical bone (CB), trabecular bone (TB), and background (BK) regions of interest (ROIs) used for contrast-to-noise ratio (CNR) and coefficient of variation (COV) measurements. Red, yellow, and white boxes indicate CB, TB, and BK ROIs, respectively.

To quantitatively assess noise reduction performance, contrast-to-noise ratio (CNR) and coefficient of variation (COV) values were calculated using manually selected ROIs within the mandibular bone. The ROIs included both cortical bone (CB) and trabecular bone (TB) regions. A fixed ROI size of 10 × 10 pixels was applied to ensure consistency across all measurements. ROIs were selected from regions with relatively consistent intensity patterns within CB and TB to ensure reliable quantitative analysis. Representative ROI placements used for quantitative evaluation are shown in Figure 2.

CNR, which evaluates the contrast between the target region and the background relative to image noise, was defined as:

(6) $$\mathrm{CNR}=\;\frac{\left|S_{M}-S_{\mathrm{BK}}\right|}{\sqrt{\sigma_{M}^{2}+\sigma_{\mathrm{BK}}^{2}}}$$

where *S_M_* and *S_BK_* represent the mean signal intensities of each ROI and background region, respectively, and *σ_M_* and *σ_BK_* represent their corresponding standard deviations. A higher CNR value indicates that the target structure is more clearly distinguishable from the background despite the presence of noise, reflecting improved image contrast and noise reduction performance.

COV, which quantifies the relative magnitude of image noise within the selected ROI, was calculated as:

(7) $$\mathrm{COV}=\;\frac{\sigma_{M}}{\mu }$$

where *σ_M_* represents the standard deviation and *μ* represents the mean intensity value within the selected ROI. A lower COV value indicates reduced intensity variation relative to the mean signal level, reflecting lower image noise and improved image uniformity.

The relative difference, which quantifies the degree of deviation in noise characteristics between the FNLM-processed and reference images, was calculated as:

(8) $$\mathrm{Relative}\;\mathrm{difference}\;\left(\%\right)=\;\frac{\left|X_{\mathrm{FNLM}}-X_{R}\right|}{X_{R}}\;\times 100$$

where *X_FNLM_* represents the CNR or COV value obtained after FNLM algorithm processing and *X_R_* represents the corresponding value measured in the reference image. A lower relative difference indicates greater agreement in noise characteristics between the FNLM-processed and reference images, reflecting a noise level more comparable to that of the original reference image.

#### 2.4.2. Similarity Evaluation Metrics

Noise-reduced images were compared with reference images using root mean square error (RMSE), peak signal-to-noise ratio (PSNR), and the structural similarity index measure (SSIM).

RMSE, which quantifies the average pixel-wise difference between the noise-reduced image and the reference image, was calculated as:

(9) $$\mathrm{RMSE}=\;\sqrt{\frac{1}{\mathrm{MN}}\sum_{i=1}^{M}\sum_{j=1}^{N}{\left(I(i,j)-R(i,j)\right)}^{2}}\;$$

where *I*(*i*,*j*) represents the pixel value of the noise-reduced image and *R*(*i*,*j*) represents the corresponding pixel value of the reference image. A lower RMSE value indicates that the noise-reduced image deviates less from the reference image, reflecting a smaller reconstruction error and greater similarity between the two images.

PSNR, which evaluates image reconstruction quality by measuring the ratio between the maximum possible pixel intensity and the residual error, was calculated as:

(10) $$\mathrm{PSNR}=\;20\;\log_{10}\left(\frac{{\mathrm{MAX}}_{I}}{\mathrm{RMSE}}\right)$$

where *MAX_I_* denotes the maximum possible pixel intensity value of the image. A higher PSNR value indicates that the noise-reduced image contains less reconstruction error relative to the reference image, reflecting better image quality and greater similarity between the two images.

SSIM, which assesses image similarity based on luminance, contrast, and structural information, was calculated as:

(11) $$\mathrm{SSIM}(x,y)=\frac{\left(2\mu_{x}\mu_{y}+C_{1})(2\sigma_{\mathrm{xy}}+C_{2}\right)}{\left(\mu_{x}^{2}+\;\mu_{y}^{2}+C_{1})(\sigma_{x}^{2}+\;\sigma_{y}^{2}+C_{2}\right)}$$

where *μ_x_* and *μ_y_* represent the mean intensity values of images *x* and *y*, respectively, $\sigma_{x}^{2}$ and $\sigma_{y}^{2}$ represent the variances, and *σ_xy_* represents the covariance between the two images. A higher SSIM value indicates that the noise-reduced image preserves the structural characteristics of the reference image more effectively, reflecting greater structural similarity between the two images.

#### 2.4.3. No-Reference Image Quality Assessment Metrics

To further evaluate perceptual image quality without requiring reference images, the blind/referenceless image spatial quality evaluator (BRISQUE) and natural image quality evaluator (NIQE) were additionally calculated.

BRISQUE, which evaluates perceptual image quality based on deviations from natural scene statistics, was calculated as:

(12) $$\hat{I}(i,j)=\;\frac{I\left(i,j\right)-\;\mu (i,j)}{\sigma \left(i,j\right)+C}$$

where *I*(*i*,*j*) represents the pixel intensity at location (*i*,*j*), *μ*(*i*,*j*) and *σ*(*i*,*j*) denote the local mean and standard deviation, respectively, and *C* is a constant used to prevent instability when the denominator approaches zero. A lower BRISQUE value indicates that the image exhibits fewer statistical distortions and is perceived to have higher image quality.

NIQE, which quantifies deviations from the statistical characteristics of natural images, was calculated as:

(13) $$D=\;\sqrt{{\left(v_{1}-v_{2}\right)}^{T}\;{\left(\frac{\sum_{1}+\sum_{2}}{2}\right)}^{-1}(v_{1}-v_{2})}$$

where *v*_1_ and *v*_2_ represent the mean feature vectors of the test image and the reference statistical model, respectively, and $\sum_{1}$ and $\sum_{2}$ represent their corresponding covariance matrices. A lower NIQE value indicates that the statistical properties of the image are closer to those of high-quality natural images, reflecting reduced perceptual distortion.

The search window size providing the best overall compromise among the evaluated conditions was identified by jointly considering the following aspects. First, agreement of noise characteristics with the reference images was assessed using the relative differences in CNR and COV defined in Equation (8). For each search window size, the four relative differences (CNR and COV in the CB and TB regions) were averaged with equal weights, and the condition with the lowest mean relative difference was regarded as showing the closest overall agreement with the reference images. Second, the search window size beyond which further increases in search window size resulted in minimal changes in the similarity evaluation metrics was identified. No-reference image quality assessment metrics were additionally used as supplementary measures to identify conditions minimizing excessive smoothing associated with noise reduction. The computational efficiency of the selected search window size was further examined by comparison of its processing time with those of the other conditions.

These criteria were not prespecified as a quantitative decision rule before the analysis. Instead, the criteria were applied after the quantitative results had been examined, and the selection therefore represents a post hoc interpretation of the results and not a predefined selection procedure.

### 2.5. Statistical Analysis

**Figure 3:**
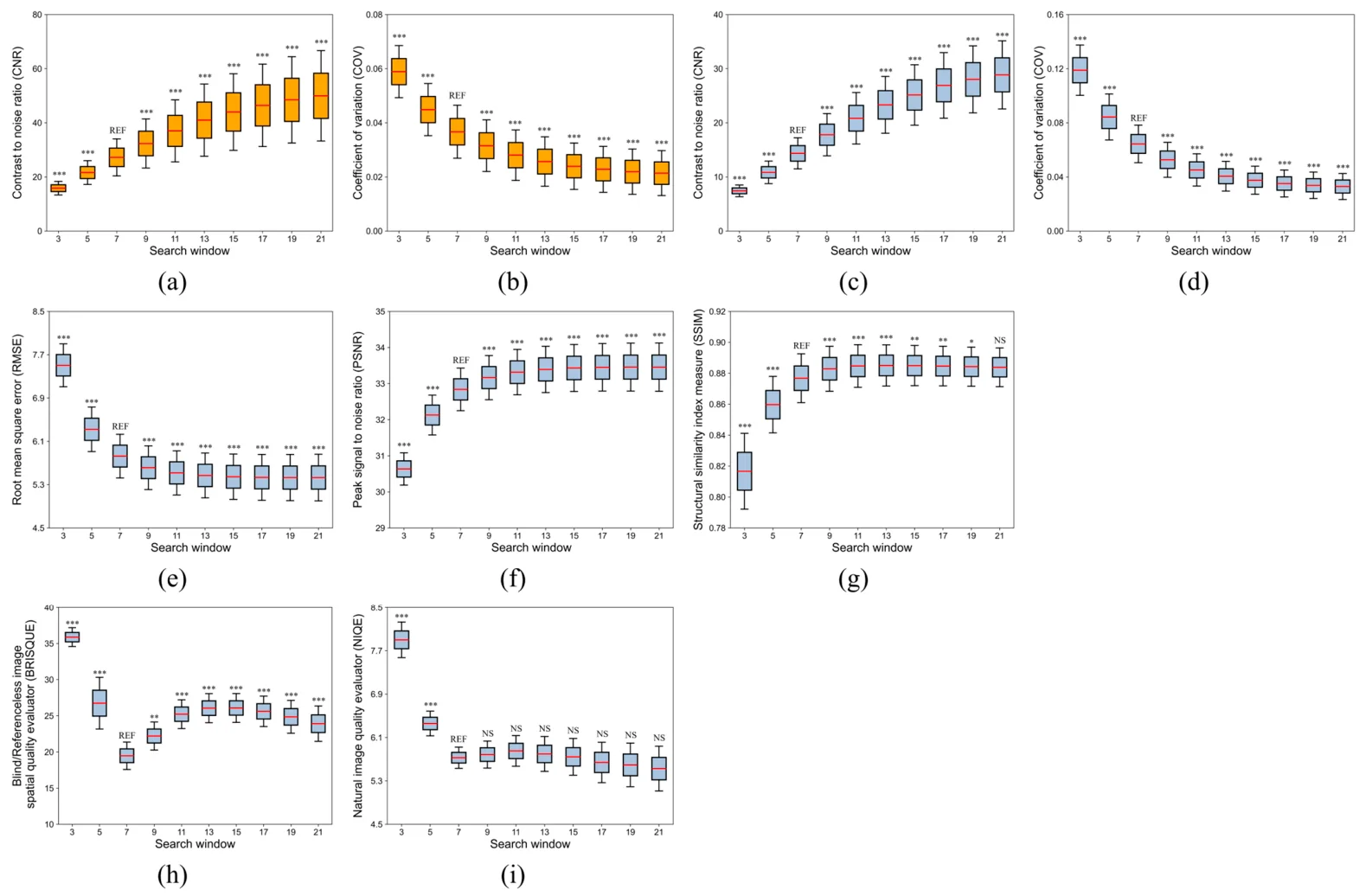
Image quality evaluation results of the fast non-local means (FNLM) algorithm as a function of search window size in noise-degraded dental cone-beam computed tomography (CBCT) images: (**a**,**b**) contrast-to-noise ratio (CNR) and coefficient of variation (COV) in the cortical bone (CB), (**c**,**d**) in the trabecular bone (TB), (**e**–**g**) root mean square error (RMSE), peak signal-to-noise ratio (PSNR), and structural similarity index measure (SSIM) for similarity evaluation, and (**h**,**i**) blind/referenceless image spatial quality evaluator (BRISQUE) and natural image quality evaluator (NIQE) for no-reference image quality assessment. REF indicates the 7 × 7 search window, which was used as the reference condition for the post hoc comparisons. Asterisks above the other search windows indicate statistically significant differences compared with the 7 × 7 search window based on Bonferroni-adjusted pairwise comparisons (one, two, and three asterisks indicate *p* < 0.05, *p* < 0.01, and *p* < 0.001, respectively); NS indicates no significant difference (*p* ≥ 0.05).

To compare performance differences across search window sizes, repeated-measures analysis of variance (RM-ANOVA) was performed using IBM SPSS Statistics software (ver. 32.0.0.0; IBM Corp., Armonk, NY, USA). Although image quality evaluation metrics inherently change with parameter variations, RM-ANOVA was used to evaluate the effects of varying search window sizes across multiple samples. The assumptions of RM-ANOVA were assessed using the Shapiro–Wilk test for normality and Mauchly’s test for sphericity. Although deviations from normality were observed for some metrics at specific search window sizes, RM-ANOVA was retained because it can provide a relatively robust approach to moderate departures from normality when the sphericity assumption is appropriately assessed and corrected. For violations of the sphericity assumption, the results were adjusted using the Greenhouse-Geisser correction to minimize potential statistical errors [28]. However, the large partial *η*^2^ values primarily reflect systematic effects associated with changes in the algorithm parameter and should not be interpreted as evidence of clinical importance by themselves. Following significant ANOVA results, Bonferroni-corrected post hoc pairwise comparisons were performed among all search window sizes, and the comparisons between the selected 7 × 7 condition and each of the other conditions are reported in Tables 3–5 and Figure 3. A *p*-value of less than 0.05 was considered statistically significant. A sensitivity analysis was performed using G*Power ((ver. 3.1.9.7; Heinrich Heine University Düsseldorf, Düsseldorf, Germany). Because preservation of structural information, in addition to noise reduction, was considered an important factor in FNLM parameter selection, SSIM was selected as the representative evaluation metric (with a total sample size of 17, 10 repeated measurements, an *α* level of 0.05, a statistical power of 0.80, an average correlation among repeated measures of 0.912, and a nonsphericity correction of *ε* = 0.113).

## 3. Results

### 3.1. Comparison of FNLM Search Window Sizes Based on Quantitative Image Quality Evaluation

Figure 3a–d and Table 1 present the CNR and COV results obtained after applying the FNLM algorithm with different search window sizes under simulated noise degradation. In both CB and TB regions, CNR increased while COV decreased as the search window size increased. RM-ANOVA confirmed statistically significant differences among search window sizes for both metrics, as summarized in Table 2 (*p* < 0.001, partial *η^2^* ≥ 0.839). The post hoc sensitivity analysis indicated that the present repeated-measures design was capable of detecting an effect size of Cohen’s *f* = 0.202 with 80% statistical power. Overall, the mean CNR ranged from 15.8651 to 49.9878 in the CB region and from 7.4323 to 28.8653 in the TB region, exhibiting an increasing trend with increasing search window size. In contrast, the mean COV ranged from 0.0589 to 0.0214 and from 0.1189 to 0.0329 in the CB and TB regions, respectively, exhibiting a decreasing trend.

As presented in Table 3, Bonferroni pairwise comparisons demonstrated that differences between the 7 × 7 condition and the other search window sizes were statistically significant for all comparisons. For CNR, all pairwise comparisons were statistically significant in both the CB and TB regions (all *p* < 0.001). Similarly, for COV, all comparisons were statistically significant in both the CB and TB regions (all *p* < 0.001). These findings indicate that larger search window sizes more effectively suppressed stochastic noise by incorporating a greater number of structurally similar neighboring patches into the weighted averaging process.

Although CNR and COV consistently improved with increasing search window size, these metrics only evaluate local noise characteristics within manually selected ROIs and therefore do not by themselves indicate an appropriate search window size. Excessively large search windows increase smoothing effects by averaging over a broader neighborhood, thereby compromising the preservation of fine anatomical structures. Accordingly, the search window sizes were compared to identify the condition that achieved an appropriate balance between noise reduction and structural preservation. To further evaluate the agreement in noise characteristics between the FNLM-processed and reference images, relative differences in CNR and COV were calculated for both the CB and TB regions. The relative differences were calculated separately for each metric at each search window size according to Equation (8), using the mean values in Table 1. Because their magnitudes varied across the four metrics (CNR and COV in the CB and TB regions), the mean relative difference was additionally calculated for each search window size by averaging the four values with equal weights, providing a single measure of overall agreement with the reference images.

The mean relative difference was lowest for the 5 × 5 condition (10.3%), followed by the 7 × 7 (15.2%), 9 × 9 (35.2%), and 3 × 3 (44.3%) conditions, and increased progressively for larger search windows, reaching 91.0% for the 21 × 21 condition. Under the 7 × 7 condition, the relative differences were 17.4% for CNR and 7.1% for COV in the CB region, and 23.0% for CNR and 13.2% for COV in the TB region. These findings indicate that the noise characteristics of the FNLM-processed images approached those of the reference images at the 5 × 5 and 7 × 7 conditions, whereas larger search windows progressively exceeded the reference level, reflecting smoothing beyond that required to restore the reference noise characteristics. Because the 5 × 5 condition showed significantly lower similarity to the reference images and higher BRISQUE values than the 7 × 7 condition (Table 4 and Table 5), the 7 × 7 condition was regarded as the smallest search window that restored reference-level noise characteristics without compromising structural similarity.

To complement the local ROI-based evaluation provided by CNR and COV, overall image similarity was additionally evaluated using RMSE, PSNR, and SSIM. As illustrated in Figure 3e–g and Table 1, the similarity metrics exhibited rapid improvement up to the 7 × 7 condition, followed by relatively stable trends across larger search window sizes. RM-ANOVA confirmed that these differences among search window sizes were statistically significant, as summarized in Table 2 (*p* < 0.001, partial *η*^2^ > 0.888). Specifically, the greatest improvements in similarity metrics were observed up to the 7 × 7 condition. The mean RMSE decreased by 22.3% from the 3 × 3 to the 7 × 7 condition, whereas only an additional 6.8% reduction was observed with larger search window sizes. Notably, the mean PSNR increased by 2.2051 dB up to the 7 × 7 condition, followed by only a further increase of 0.6167 dB. Meanwhile, the mean SSIM increased by 0.0601 up to the 7 × 7 condition, whereas the increase beyond this condition was limited to only 0.0071.

As presented in Table 4, Bonferroni pairwise comparisons demonstrated statistically significant differences between the 7 × 7 condition and most alternative search window sizes. Significant differences were also observed for all RMSE and PSNR comparisons (all *p* < 0.001). Similarly, SSIM differed significantly between the 7 × 7 condition and all other search window sizes except the 21 × 21 condition (*p* > 0.050), with statistical significance observed for all remaining pairwise comparisons (all *p* ≤ 0.030). These findings indicate that most improvements in similarity to the reference images were already achieved under the 7 × 7 condition. Beyond this point, only marginal additional improvements in RMSE, PSNR, and SSIM were observed despite further increases in search window size, indicating that larger search windows provided minimal additional benefit.

To further evaluate the effect of smoothing associated with larger search window sizes on perceptual image quality, no-reference image quality was additionally assessed using BRISQUE and NIQE. As illustrated in Figure 3h,i and Table 1, both no-reference metrics showed marked improvement up to the 7 × 7 condition, followed by relatively small changes with larger search window sizes. Specifically, the mean BRISQUE decreased by 45.7% between the 3 × 3 and 7 × 7 conditions and subsequently increased as the search window size became larger. Similarly, the mean NIQE decreased by 27.5% between the 3 × 3 and 7 × 7 conditions, with only minor variations observed at larger search window sizes. RM-ANOVA confirmed that these differences among search window sizes were statistically significant, as summarized in Table 2 (*p* < 0.001, partial *η*^2^ > 0.730).

As presented in Table 5, Bonferroni pairwise comparisons demonstrated statistically significant differences between the 7 × 7 condition and most alternative search window sizes. BRISQUE showed significant differences for all pairwise comparisons involving the 7 × 7 condition (all *p* ≤ 0.003). In contrast, NIQE showed no statistically significant differences between the 7 × 7 condition and larger search window sizes (all *p* = 1.000), indicating that only minimal changes were observed beyond the 7 × 7 condition. Collectively, the no-reference image quality metrics indicated that most perceptual image quality improvements were achieved under the 7 × 7 condition, whereas larger search window sizes provided little additional benefits.

**Figure 4:**
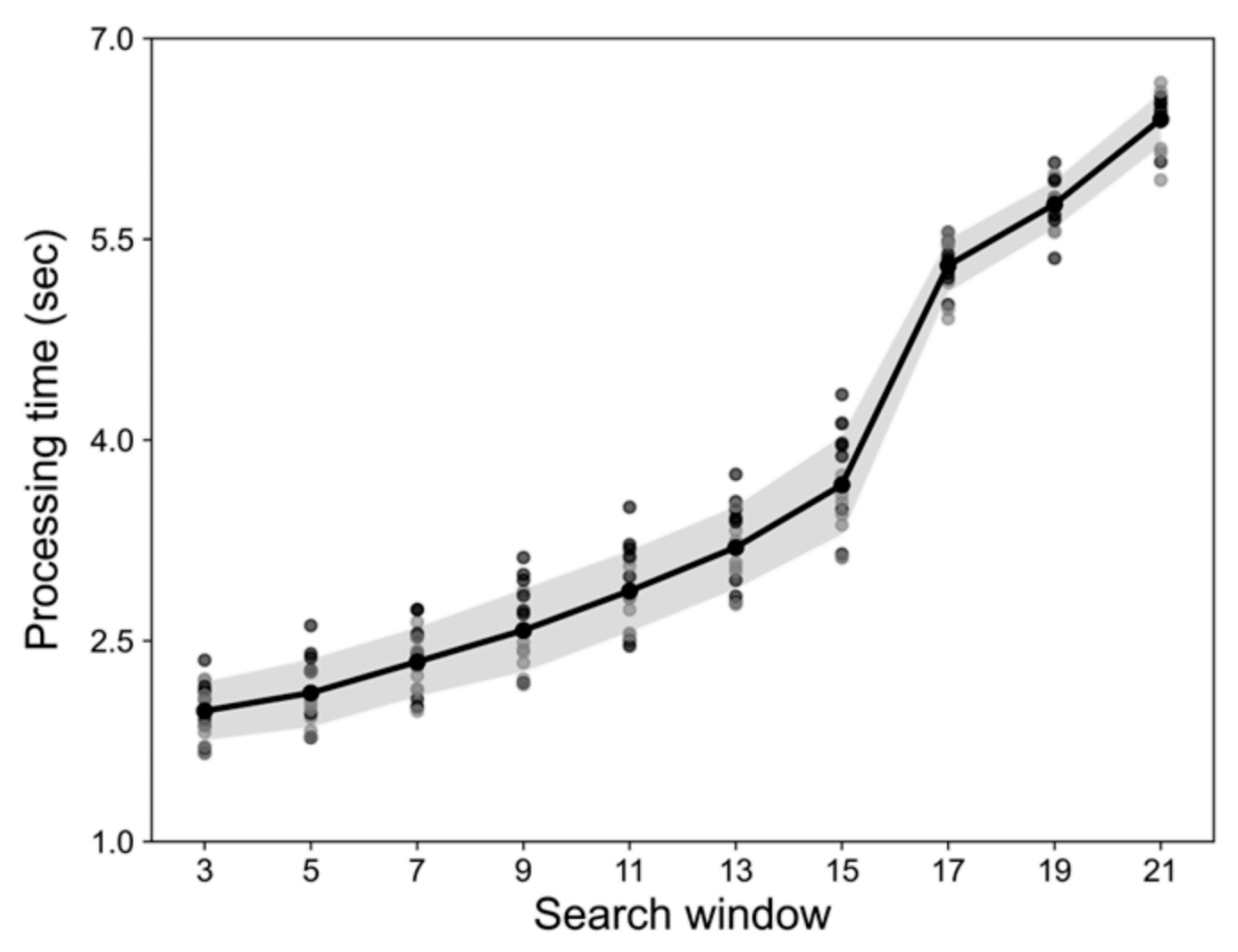
Changes in processing time of the fast non-local means (FNLM) algorithm as the search window size increased. Dots indicate the processing times of the individual axial slices (n = 17), the solid line indicates the mean processing time, and the shaded area indicates ±1 standard deviation.

To additionally assess the computational cost associated with varying search window sizes, the processing time of the FNLM algorithm was measured on the workstation described in Section 2.3. For each search window size, the processing time was measured for each of the 17 selected axial slices, and the measured time included only the FNLM filtering step. The mean processing time across the 17 slices was used as the corresponding value in Figure 4. Specifically, the mean values increased from 1.98 ± 0.22 s for the 3 × 3 condition to 2.34 ± 0.26 s for the 7 × 7 condition and 6.40 ± 0.20 s for the 21 × 21 condition. As shown in Figure 4, processing time increased progressively with increasing search window size, with a more pronounced increase observed from the 17 × 17 condition onward. Given the limited improvement in image quality observed beyond the 7 × 7 condition, the balance between image quality improvements and computational cost favored the 7 × 7 search window.

### 3.2. Visual Evaluation of FNLM Noise Reduction According to Search Window Size

**Figure 5:**
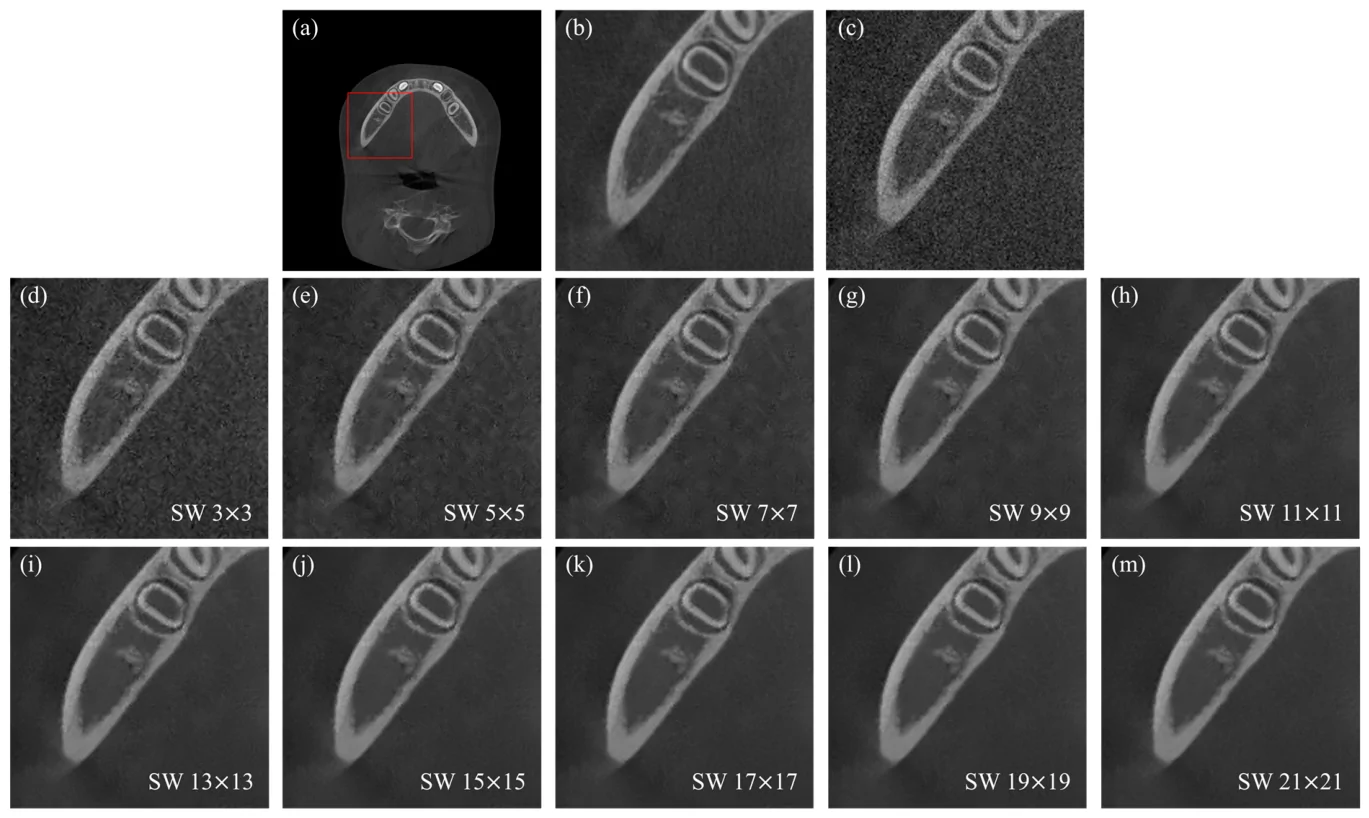
Magnified region results of the fast non-local means (FNLM) algorithm applied to cone-beam computed tomography (CBCT) images degraded with simulated noise: (**a**) reference axial slice image with the region used for visual evaluation indicated by a red box, (**b**) reference image, (**c**) noise-degraded image, and (**d**–**m**) FNLM noise-reduced images using search window (SW) sizes ranging from 3 × 3 to 21 × 21.

Visual evaluation was performed to visually assess the image characteristics associated with the selected search window size identified through the quantitative analyses. Figure 5 presents the results of applying ten different search window sizes, ranging from 3 × 3 to 21 × 21, to CBCT images degraded with simulated noise (*σ* = 0.05). The 7 × 7 search window provided an appropriate balance between noise reduction and preservation of anatomical structures. Although increasing the search window size progressively improved suppression of the added noise, search window sizes larger than 7 × 7 produced increasingly smooth images with reduced visibility of fine trabecular structures and blurred anatomical boundaries compared to the reference image. These visual observations indicate that the additional noise reduction achieved with larger search window sizes was accompanied by over-smoothing, supporting the selection of the 7 × 7 search window as the best overall compromise among the evaluated conditions.

## 4. Discussion

Mandibular fractures represent a substantial proportion of pediatric maxillofacial injuries. CBCT provides essential 3D information for evaluating complex fracture patterns and anatomical structures. Although low-dose imaging protocols are recommended for pediatric patients owing to their increased radiosensitivity, the elevated noise associated with dose reduction can impair the visualization of fine anatomical details and potentially affect image interpretability. To address this limitation, various post-processing techniques have been investigated [29,30,31,32]. Among these, the FNLM algorithm effectively suppresses noise while preserving anatomical structures by exploiting non-local self-similarity within the image. In particular, the search window size is a critical parameter that influences the balance between noise reduction and structural preservation. Therefore, this study applied FNLM to pediatric CBCT images degraded by simulated noise and compared different search window sizes to identify the conditions that provided the most balanced trade-off between noise reduction and structural preservation in the mandibular region.

To compare search window sizes, a quantitative evaluation of noise level, similarity to the reference images, and no-reference image quality was performed. The addition of simulated noise resulted in substantial image degradation, with mean CNR values decreasing to 10.1467 and 4.5804 in the CB and TB regions, respectively. Concurrently, mean COV values increased to 0.0826 and 0.1728 compared to the reference images. Based on a comprehensive analysis of the quantitative evaluation results, the 7 × 7 search window represented the best overall compromise among the evaluated search window sizes under the simulated conditions of this study. Under this condition, the noise characteristics of the processed images were close to those of the reference images, with a mean relative difference of 15.2%, second only to the 5 × 5 condition, which showed significantly lower similarity to the reference images and lower perceptual image quality. The similarity metrics showed substantial improvements up to the 7 × 7 condition, with no meaningful additional gains observed thereafter. Notably, even when the search window was expanded to the largest evaluated size (21 × 21), the difference in SSIM compared to the 7 × 7 condition failed to reach statistical significance (*p* = 0.071). The no-reference image quality measures showed the lowest BRISQUE value at 7 × 7, while NIQE was not significantly different from that of larger search window conditions, suggesting that excessive smoothing may have been minimized while achieving sufficient noise reduction and supporting the selection of 7 × 7 as the best overall compromise. Although larger search windows provided further improvements in some noise-related metrics, improvements in overall image quality were limited beyond a certain window size, whereas processing time continued to increase with increasing search window size. Thus, the smallest search window that achieved sufficient image quality was considered the most reasonable choice for computational efficiency, with the 7 × 7 search window selected for FNLM processing under the conditions evaluated in this study.

This study evaluated, both quantitatively and visually, the effects of FNLM according to search window size on noise reduction performance using noise-degraded pediatric CBCT images derived from a publicly available dataset. However, several limitations should be considered. First, the present study was conducted using CBCT data from a relatively small sample of 17 pediatric patients. In addition, although the MMDental dataset included CBCT data acquired using two different imaging systems, scanner-specific information for individual datasets was unavailable. Although differences in scanner hardware and acquisition characteristics may influence baseline image properties and noise characteristics, the primary objective of the present study was to evaluate the relative effects of FNLM search window size under standardized noise simulation conditions rather than to compare image quality between different CBCT systems. Second, the noise-degraded images were generated by adding synthetic noise, and because the Poisson component generated under the present implementation was negligible, the resulting degradation was predominantly Gaussian. Consequently, the simulation did not reproduce the signal-dependent quantum noise of true low-dose CBCT acquisition and represented only a partial approximation of the physical acquisition conditions of true low-dose CBCT imaging. The Gaussian noise level (*σ* = 0.05) was selected by a single researcher based on visual assessment to produce a sufficiently degraded imaging condition while maintaining recognizable cortical and trabecular bone structures. Other noise levels were not quantitatively evaluated, which may limit the assessment of FNLM performance across different levels of simulated image degradation. Furthermore, under clinical low-dose conditions, increased image noise is accompanied by incomplete acquisition of fine anatomical information, including TB structures. Because post-processing algorithms such as FNLM can enhance only the information contained within the acquired images, anatomical details lost during image acquisition cannot be recovered [33,34,35]. Accordingly, the preservation of fine anatomical structures observed in the present study should be interpreted within the context of the simulated noise degradation. Third, this study was conducted using CBCT data from pediatric patients without mandibular fractures. Therefore, evaluating the impact of FNLM processing on the visualization of complex fracture lines and diagnostic performance in traumatic cases was beyond the scope of the present study. To establish the generalizability of the present findings, future studies should incorporate larger and more diverse patient populations, well-documented scanner and acquisition parameters, clinically acquired low-dose CBCT data, a physically calibrated signal-dependent noise model, and clinically relevant pathological conditions. Such future studies would enable validation of the selected FNLM parameters across different imaging environments and further assessment of their ability to preserve diagnostically important anatomical information under actual low-dose and pathological conditions.

Despite these limitations, the present study has significance in that it systematically analyzed the influence of FNLM search window size on image quality and quantitatively identified the condition that provided the best overall compromise between noise reduction and anatomical structure preservation among the evaluated search window sizes. Deep learning-based approaches have emerged as prominent methods for improving image quality in low-dose imaging; however, these methods generally require model training. In contrast, FNLM can be directly applied without requiring prior training and adapted through adjustment of interpretable parameters [36,37]. Considering the substantial anatomical variability among individuals in the oral and maxillofacial region and the diversity of diagnostic tasks, parameter adjustment may represent a practical approach.

The quantitative comparison strategy established in the present study provides a foundation for parameter selection of FNLM in pediatric mandibular CBCT imaging. Although the smoothing factor and patch size are also important parameters of the FNLM algorithm, the present study prioritized the search window because it directly influences both noise reduction performance and computational efficiency. The search window determines the spatial region over which similar image patches are searched, such that enlarging the search window substantially increases the number of candidate patches evaluated during filtering and, consequently, the computational cost [15,38]. In the present study, although image quality metrics, including noise level and image similarity, continued to improve with increasing search window size, the incremental improvements in noise reduction performance became marginal beyond the 7 × 7 condition, whereas processing time increased steadily with larger search windows. These findings suggest that appropriate selection of the search window, which affects noise reduction performance, structural preservation, and computational efficiency, is a practical approach to balancing these factors in FNLM. Based on this approach, future studies could further investigate combinations of FNLM parameters through additional parameter adjustments for improving image quality in pediatric CBCT imaging, while extending the application of FNLM-based image quality enhancement to a broader range of dental imaging data beyond CBCT.

Beyond the potential applicability of FNLM parameter selection discussed above, this study provides additional methodological value by establishing a comprehensive quantitative framework through the integration of no-reference image quality assessment. In contrast to previous studies, which primarily evaluated the noise reduction performance of NLM-based algorithms using conventional noise and similarity metrics, the present study incorporated no-reference image quality assessment using BRISQUE and NIQE into the parameter comparison process [39,40]. This was crucial, because excessive smoothing caused by large search window sizes is not fully reflected by local noise level and reference-based similarity metrics alone. The use of no-reference image quality assessment has been reported across various medical imaging modalities and has also been extended to dental imaging such as dental panoramic X-ray [41,42]. However, caution is warranted when interpreting no-reference image quality scores in dental imaging. Metrics such as BRISQUE and NIQE were developed based on statistical characteristics associated with natural images and may not fully reflect the preservation of diagnostically relevant anatomical structures in dental radiographic images. Therefore, a lower BRISQUE or NIQE score should not be interpreted as direct evidence of improved diagnostic quality. In the present study, BRISQUE and NIQE were thus interpreted as complementary measures of perceptual image quality rather than as independent indicators of diagnostic image quality. Their combined interpretation with conventional noise- and similarity-based metrics enabled a more balanced assessment of noise suppression and anatomical structure preservation. Future studies should further investigate the relationships between these metrics and expert-based or task-specific assessments to support more robust selection of FNLM parameters and a comprehensive quantitative framework for evaluating image enhancement algorithms in dental CBCT imaging.

The quantitative comparison established in this study provides a systematic framework for the selection of appropriate FNLM parameters by balancing noise reduction, preservation of anatomical structures, perceptual image quality, and computational efficiency. This approach may facilitate the development of image enhancement protocols for low-dose CBCT imaging, particularly in pediatric patients, where radiation dose reduction and diagnostic image quality must be carefully balanced. Although further validation using clinically acquired low-dose CBCT datasets and trauma cases is required, the present findings provide quantitative evidence supporting FNLM parameter selection and represent an initial step toward establishing image enhancement strategies for pediatric mandibular assessment in dental CBCT imaging.

## 5. Conclusions

In this study, pediatric CBCT images were degraded by adding simulated noise to assess the effects of FNLM search window size on image quality. Increasing the search window size enhanced noise reduction performance; however, improvements in reference-image similarity and perceptual image quality plateaued beyond the 7 × 7 condition. Among the evaluated conditions, a 7 × 7 search window provided the best overall compromise among stochastic noise reduction, preservation of trabecular microarchitecture, and similarity to the reference image. These findings provide objective evidence supporting the potential of FNLM-based image enhancement for improving image quality in noise-degraded pediatric CBCT images and demonstrate the value of systematic parameter selection within the simulated conditions evaluated in this study.

## Figures and Tables

**Table 1:** Quantitative image quality evaluation results according to fast non-local means (FNLM) search window size (mean ± standard deviation).

	Reference	Noisy	Search Window 3 × 3	Search Window 5 × 5	Search Window 7 × 7	Search Window 9 × 9	Search Window 11 × 11	Search Window 13 × 13	Search Window 15 × 15	Search Window 17 × 17	Search Window 19 × 19	Search Window 21 × 21
CNR (CB)	23.2015 ± 3.6396	10.1467 ± 1.0461	15.8651 ± 2.5001	21.6298 ± 4.3950	27.2378 ± 6.8411	32.3629 ± 9.0699	37.0377 ± 11.4497	41.0051 ± 13.3425	44.0065 ± 14.1583	46.4610 ± 15.2250	48.5055 ± 15.9374	49.9878 ± 16.7088
COV (CB)	0.0395 ± 0.0060	0.0826 ± 0.0092	0.0589 ± 0.0096	0.0449 ± 0.0096	0.0367 ± 0.0098	0.0316 ± 0.0095	0.0281 ± 0.0093	0.0257 ± 0.0091	0.0240 ± 0.0086	0.0228 ± 0.0085	0.0220 ± 0.0084	0.0214 ± 0.0083
CNR (TB)	11.6806 ± 1.2800	4.5804 ± 0.4779	7.4323 ± 1.0782	10.8544 ± 2.0804	14.3621 ± 2.8738	17.8037 ± 3.9007	20.8479 ± 4.7506	23.3235 ± 5.2533	25.1495 ± 5.5830	26.9234 ± 6.0621	28.0325 ± 6.1861	28.8653 ± 6.2905
COV (TB)	0.0742 ± 0.0066	0.1728 ± 0.0169	0.1189 ± 0.0186	0.0844 ± 0.0170	0.0644 ± 0.0139	0.0527 ± 0.0129	0.0452 ± 0.0119	0.0405 ± 0.0110	0.0376 ± 0.0103	0.0352 ± 0.0099	0.0338 ± 0.0098	0.0329 ± 0.0096
RMSE	-	11.0553 ± 0.2463	7.5049 ± 0.3964	6.3229 ± 0.4109	5.8278 ± 0.4037	5.6140 ± 0.4035	5.5174 ± 0.4083	5.4703 ± 0.4139	5.4463 ± 0.4190	5.4345 ± 0.4233	5.4313 ± 0.4272	5.4320 ± 0.4303
PSNR	-	27.2614 ± 0.1917	30.6353 ± 0.4486	32.1298 ± 0.5524	32.8404 ± 0.5389	33.1665 ± 0.6113	33.3184 ± 0.6284	33.3940 ± 0.6415	33.4329 ± 0.6513	33.4522 ± 0.6586	33.4579 ± 0.6643	33.4571 ± 0.6686
SSIM	-	0.6765 ± 0.0362	0.8167 ± 0.0245	0.8597 ± 0.0183	0.8768 ± 0.0158	0.8829 ± 0.0145	0.8847 ± 0.0138	0.8851 ± 0.0133	0.8850 ± 0.0130	0.8847 ± 0.0128	0.8843 ± 0.0126	0.8839 ± 0.0125
BRISQUE	17.6518 ± 2.3060	44.2911 ± 0.5481	35.8791 ± 1.3028	26.7489 ± 3.5740	19.4753 ± 1.8907	22.2112 ± 1.9344	25.2269 ± 1.9905	26.0584 ± 2.0142	26.0860 ± 1.9960	25.6214 ± 2.1087	24.8617 ± 2.2723	23.9147 ± 2.4413
NIQE	3.3400 ± 0.2970	10.9984 ± 0.7075	7.9004 ± 0.3279	6.3586 ± 0.2276	5.7277 ± 0.1950	5.7859 ± 0.2492	5.8529 ± 0.2830	5.7968 ± 0.3218	5.7440 ± 0.3389	5.6410 ± 0.3723	5.5953 ± 0.4012	5.5274 ± 0.4129

CNR, contrast-to-noise ratio; CB, cortical bone; COV, coefficient of variation; TB, trabecular bone; RMSE, root mean square error; PSNR, peak signal-to-noise ratio; SSIM, structural similarity index measure; BRISQUE, blind/referenceless image spatial quality evaluator; NIQE, natural image quality evaluator.

**Table 2:** Repeated-measures analysis of variance (RM-ANOVA) results for image quality metrics according to fast non-local means (FNLM) search window size.

Metrics	*F*	df (GG)	*ε*	*p*-Value	Partial *η*^2^
CNR (CB)	83.68	9 (1.062)	0.118	<0.001	0.839
COV (CB)	566.94	9 (1.417)	0.157	<0.001	0.973
CNR (TB)	201.96	9 (1.284)	0.143	<0.001	0.927
COV (TB)	381.47	9 (1.274)	0.142	<0.001	0.960
RMSE	9248.97	9 (1.397)	0.155	<0.001	0.998
PSNR	1944.78	9 (1.057)	0.117	<0.001	0.992
SSIM	126.24	9 (1.017)	0.113	<0.001	0.888
BRISQUE	43.31	9 (2.782)	0.309	<0.001	0.730
NIQE	204.42	9 (1.766)	0.196	<0.001	0.927

df, degrees of freedom; GG, greenhouse-geisser; CNR, contrast-to-noise ratio; CB, cortical bone; COV, coefficient of variation; TB, trabecular bone; RMSE, root mean square error; PSNR, peak signal-to-noise ratio; SSIM, structural similarity index measure; BRISQUE, blind/referenceless image spatial quality evaluator; NIQE, natural image quality evaluator.

**Table 3:** Bonferroni post hoc pairwise comparisons of noise level evaluation metrics with the 7 × 7 search window as the reference condition.

Metrics	Comparison	MD (95% CI)	*p*-Value
CNR (CB)	3 × 3	11.373 (6.806 to 15.940)	<0.001
	5 × 5	5.608 (3.094 to 8.121)	<0.001
	9 × 9	−5.125 (−7.576 to −2.674)	<0.001
	11 × 11	−9.800 (−14.818 to −4.782)	<0.001
	13 × 13	−13.767 (−20.550 to −6.985)	<0.001
	15 × 15	−16.769 (−24.216 to −9.321)	<0.001
	17 × 17	−19.223 (−27.658 to −10.789)	<0.001
	19 × 19	−21.268 (−30.400 to −12.136)	<0.001
	21 × 21	−22.750 (−32.682 to −12.819)	<0.001
COV (CB)	3 × 3	−0.022 (−0.026 to −0.018)	<0.001
	5 × 5	−0.008 (−0.010 to −0.007)	<0.001
	9 × 9	0.005 (0.004 to 0.006)	<0.001
	11 × 11	0.009 (0.007 to 0.010)	<0.001
	13 × 13	0.011 (0.009 to 0.013)	<0.001
	15 × 15	0.013 (0.010 to 0.015)	<0.001
	17 × 17	0.014 (0.011 to 0.016)	<0.001
	19 × 19	0.015 (0.012 to 0.018)	<0.001
	21 × 21	0.015 (0.012 to 0.018)	<0.001
CNR (TB)	3 × 3	6.930 (5.010 to 8.850)	<0.001
	5 × 5	3.508 (2.561 to 4.454)	<0.001
	9 × 9	−3.442 (−4.651 to −2.232)	<0.001
	11 × 11	−6.486 (−8.635 to −4.337)	<0.001
	13 × 13	−8.961 (−11.752 to −6.171)	<0.001
	15 × 15	−10.787 (−14.006 to −7.569)	<0.001
	17 × 17	−12.561 (−16.311 to −8.812)	<0.001
	19 × 19	−13.670 (−17.633 to −9.708)	<0.001
	21 × 21	−14.503 (−18.508 to −10.499)	<0.001
COV (TB)	3 × 3	−0.055 (−0.064 to −0.045)	<0.001
	5 × 5	−0.020 (−0.025 to −0.015)	<0.001
	9 × 9	0.012 (0.008 to 0.015)	<0.001
	11 × 11	0.019 (0.014 to 0.024)	<0.001
	13 × 13	0.024 (0.018 to 0.030)	<0.001
	15 × 15	0.027 (0.020 to 0.034)	<0.001
	17 × 17	0.029 (0.022 to 0.037)	<0.001
	19 × 19	0.031 (0.023 to 0.039)	<0.001
	21 × 21	0.031 (0.023 to 0.040)	<0.001

MD, mean difference; CI, confidence interval; CNR, contrast-to-noise ratio; CB, cortical bone; COV, coefficient of variation; TB, trabecular bone.

**Table 4:** Bonferroni post hoc pairwise comparisons of similarity evaluation metrics with the 7 × 7 search window as the reference condition.

Metrics	Comparison	MD (95% CI)	*p*-Value
RMSE	3 × 3	−1.677 (−1.741 to −1.613)	<0.001
	5 × 5	−0.495 (−0.515 to −0.475)	<0.001
	9 × 9	0.214 (0.206 to 0.222)	<0.001
	11 × 11	0.310 (0.297 to 0.323)	<0.001
	13 × 13	0.357 (0.339 to 0.376)	<0.001
	15 × 15	0.381 (0.358 to 0.405)	<0.001
	17 × 17	0.393 (0.365 to 0.421)	<0.001
	19 × 19	0.396 (0.364 to 0.429)	<0.001
	21 × 21	0.396 (0.360 to 0.432)	<0.001
PSNR	3 × 3	2.205 (2.041 to 2.369)	<0.001
	5 × 5	0.711 (0.665 to 0.757)	<0.001
	9 × 9	−0.326 (−0.350 to −0.302)	<0.001
	11 × 11	−0.478 (−0.520 to −0.436)	<0.001
	13 × 13	−0.554 (−0.609 to −0.498)	<0.001
	15 × 15	−0.592 (−0.658 to −0.526)	<0.001
	17 × 17	−0.612 (−0.685 to −0.538)	<0.001
	19 × 19	−0.617 (−0.697 to −0.537)	<0.001
	21 × 21	−0.617 (−0.701 to −0.532)	<0.001
SSIM	3 × 3	0.060 (0.048 to 0.072)	<0.001
	5 × 5	0.017 (0.013 to 0.021)	<0.001
	9 × 9	−0.006 (−0.008 to −0.004)	<0.001
	11 × 11	−0.008 (−0.012 to −0.004)	<0.001
	13 × 13	−0.008 (−0.013 to −0.003)	<0.001
	15 × 15	−0.008 (−0.014 to −0.002)	0.002
	17 × 17	−0.008 (−0.014 to −0.001)	0.009
	19 × 19	−0.007 (−0.014 to 0.000)	0.030
	21 × 21	−0.007 (−0.014 to 0.000)	0.071

MD, mean difference; CI, confidence interval; RMSE, root mean square error; PSNR, peak signal-to-noise ratio; SSIM, structural similarity index measure.

**Table 5:** Bonferroni post hoc pairwise comparisons of no-reference image quality assessment metrics with the 7 × 7 search window as the reference condition.

Metrics	Comparison	MD (95% CI)	*p*-Value
BRISQUE	3 × 3	−16.404 (−19.085 to −13.723)	<0.001
	5 × 5	−7.274 (−11.745 to −2.802)	<0.001
	9 × 9	−2.736 (−4.786 to −0.686)	0.003
	11 × 11	−5.752 (−8.269 to −3.234)	<0.001
	13 × 13	−6.583 (−9.182 to −3.984)	<0.001
	15 × 15	−6.611 (−9.153 to −4.069)	<0.001
	17 × 17	−6.146 (−8.658 to −3.634)	<0.001
	19 × 19	−5.386 (−7.971 to −2.801)	<0.001
	21 × 21	−4.439 (−7.151 to −1.728)	<0.001
NIQE	3 × 3	−2.173 (−2.401 to −1.944)	<0.001
	5 × 5	−0.631 (−0.798 to −0.464)	<0.001
	9 × 9	−0.058 (−0.212 to 0.095)	1.000
	11 × 11	−0.125 (−0.347 to 0.096)	1.000
	13 × 13	−0.069 (−0.320 to 0.181)	1.000
	15 × 15	−0.016 (−0.290 to 0.257)	1.000
	17 × 17	0.087 (−0.207 to 0.380)	1.000
	19 × 19	0.132 (−0.182 to 0.447)	1.000
	21 × 21	0.200 (−0.130 to 0.531)	1.000

MD, mean difference; CI, confidence interval; BRISQUE, blind/referenceless image spatial quality evaluator; NIQE, natural image quality evaluator.

## Data Availability

Original data used in this study are available from the publicly accessible MMDental dataset. The dataset analyzed in the present study is available upon request.

## Abbreviations

The following abbreviations are used in this manuscript:

1D	One-dimensional
2D	Two-dimensional
3D	Three-dimensional
BK	Background
BRISQUE	Blind/referenceless image spatial quality evaluator
CB	Cortical bone
CBCT	Cone-beam computed tomography
CI	Confidence interval
CNR	Contrast-to-noise ratio
COV	Coefficient of variation
FNLM	Fast non-local means
IL-NIQE	Integrated local natural image quality evaluator
MD	Mean difference
NIQE	Natural image quality evaluator
NLM	Non-local means
PIQE	Perception-based image quality evaluator
PSNR	Peak signal-to-noise ratio
RM-ANOVA	Repeated-measures analysis of variance
RMSE	Root mean square error
ROI	Region of interest
SPSS	Statistical Package for the Social Sciences
SSIM	Structural similarity index measure
SW	Search window
TB	Trabecular bone
